# Clinicopathological and prognostic value of Ki-67 expression in oral malignant melanoma: A systematic review and meta-analysis

**DOI:** 10.34172/joddd.2022.024

**Published:** 2022-11-15

**Authors:** Mohammad Hossein Moltajaei, Solmaz Pourzare Mehrbani, Paria Motahari, Ramin Rezapour

**Affiliations:** ^1^Dental Student, Faculty of Dentistry, Tabriz University of Medical Sciences, Tabriz, Iran; ^2^Department of Oral Medicine, Faculty of Dentistry, Tabriz University of Medical Sciences, Tabriz, Iran; ^3^Tabriz Health Services Management Research Center, Tabriz University of Medical Sciences, Tabriz, Iran

**Keywords:** Ki-67, Meta-analysis, Oral malignant melanoma, Prognosis

## Abstract

**Background.** Ki-67 is one of the new biological markers with clinical value in the pathology and prognosis of oral melanoma. It is a nuclear protein involved in regulating cell proliferation. Some studies have suggested an association between Ki-67 and poor survival in patients with oral melanoma. This systematic review was undertaken to clarify this issue.

**Methods.** Databases of PubMed, Scopus, and Web of Science were searched using relevant English keywords from 1988 to April 2022. STATA software version 16 and random models were used for meta-analysis.

**Results.** Eleven articles were included in this systematic review, six of which were selected for meta-analysis. The mean expression of the Ki-67 index in patients with oral melanoma was estimated at 43.81% (28.66‒58.95 with 95% CI, I^2^=94.2, *P*<0.001). In addition, the results showed a significant relationship between Ki-67 expression and the prognosis of oral melanoma lesions. Increased expression of this marker weakens the prognosis and decreases the survival rate.

**Conclusion.** High expression of Ki-67 may serve as a predictive biomarker for poor prognosis in patients with malignant oral melanoma. Therefore, classifying this malignancy by Ki-67 expression may be considered for therapy regimen selection and integrated management.

## Introduction

 Melanoma is a neoplasm with various histological characteristics that accounts for 3% of all malignant tumors in the body.^1^ This tumor originates from melanocytes in the skin and oral epithelium.^2^ It is uncommon on the skin but has the poorest prognosis of oral malignancies and accounts for 0.2‒0.8% of all melanoma cases.^3^ The cause of cutaneous melanoma is sunlight; however, the cause of oral melanoma and the exact process of its formation is unknown.^4^ Oral malignant melanoma (OMM) is more invasive than skin cancers and is more likely to spread to other regions of the body and relapse after treatment.^5^ Its survival rate has been reported to be 15‒20%, compared to 67‒77% for skin types on the head and neck.^6-8^ A pigmented swelling is the earliest symptom of oral melanoma. An early phase exists in this form of melanoma. The radial development phase is followed by the invasion of the underlying layers, referred to as the “deep growth phase.”^9^

 The rate of a tumor’s proliferative activity is connected to its aggressiveness and metastatic potential. Ki-67 is a proliferative marker present in cell growth or cell division during the G1, S, G2, and M stages of the cell cycle, and its increased expression in cells implies a surge in cell proliferation.^10^ In previous research on various malignancies of the body, particularly breast^11-13^ and uterine cancers,^14^ this component has been discovered as a factor in determining the prognosis of the lesion. In some studies, Ki-67 has been used as a diagnostic factor for melanoma and benign nevi.^15^ Ki-67 has been studied as a factor in determining prognosis in various subtypes of melanoma in recent years.^16-19^ There are differing opinions in studies on the relationship between Ki-67 expression and melanoma prognosis. Some studies have reported a direct relationship between Ki-67 expression and the progression of oral melanoma, higher disease rates, and increased mortality.^10^ Other studies have associated Ki-67 expression with melanoma thickness.^20^ Väisänen et al^16^ found a significant relationship between Ki-67 expression and tumor thickness. Henrique et al^21^ reported that the Ki-67 index was positively correlated with tumor thickness and degree of tumor malignancy and poorly correlated with overall survival rate. Some of these tumors, known as the depigmented type, do not release melanin clinically or microscopically.^22^ The absence of melanin pigment release in the skin type of melanoma implies that it is aggressive.^23^ The amelanotic nature of the lesion in the oral type has not been well examined. Soares et al^24^ reported that elevated Ki-67 levels in non-pigmented melanomas also suggested a more aggressive condition.

 Based on the previous research discussed above, there is no consensus on the potential function of Ki-67 factor expression in different stages of oral melanoma for early diagnosis and prognosis. No systematic review is available on the association between Ki-67 expression and early detection and prognosis of oral melanoma. Therefore, we decided to plan a systematic review in this regard.

## Methods

 This systematic review and meta-analysis was conducted based on PICO (patient, intervention, comparison, and outcome) criteria in 2022. The following were the PICO items: patients with oral melanoma (P), Ki-67 expression gene (I), healthy people (C), oral melanoma diagnosis, and prognosis (O). The study design followed the Preferred Reporting Items for Systematic Reviews and Meta-Analyses (PRISMA) guidelines.^25^

###  Literature search

 Primarily, keywords were obtained from (MeSH) and initial literature reviews and were finalized through a pilot search. The search was conducted until April 2022.

 PubMed, Scopus, EMBASE, Cochrane Library, and Web of Science databases were searched using keywords (oral melanomas, mucosal melanoma, pigmented nodular melanoma, nonpigmented nodular melanoma, pigmented macular melanoma, pigmented mixed melanoma, nonpigmented mixed melanoma, primary oral melanoma, oral melanotic lesion, melanotic neoplasm, malignant melanoma, amelanotic oral melanoma, conventional oral melanoma, mucosal melanoma, Ki-67, Ki-67 proliferation index) connected with OR and AND. Furthermore, the references of the included articles were reviewed in addition to a manual search of similar journals to boost the search.

###  Screening and data extraction

 The Endnote X9 software was used to organize and screen obtained studies. Duplicates were eliminated first, and then the articles were screened by looking at their titles to eliminate those that were not relevant. The abstracts were reviewed, and some articles were excluded because they did not report oral or mucosal melanomas. The remaining full texts were studied, and final articles were included based on inclusion criteria. The screening process was carried out independently by two authors (MM and RR), and disagreements were solved by the third one (PM). The data from the included studies were extracted using an extraction table. The content of the extracted data was the name of the author/s, study design, sample size, age, Ki-67 expression, and results.

###  Inclusion criteria

- Studies published up to April 2022
- Articles published in English
- Articles consistent with the objectives of the study

###  Exclusion criteria

- Studies on animal specimens
- Low-quality articles (by completing quality checklists based on scoring)

###  Critical appraisal of included articles

 Critical appraisal was carried out for included articles in the meta-analysis. The studies reporting quality were independently assessed by two investigators (MM and RR) according to the 22-item STROBE checklist.^26,27^ This checklist has 22 questions. Four items related to case-control and cohort studies were deleted due to the cross-sectional nature of most papers reviewed, and the remaining 18 questions were evaluated. Checklists had a minimum score of 0 and a maximum value of 36. Good [25‒36] quality studies, medium [13‒24] quality studies, and low [0‒12] quality studies were classified.

###  Data analysis

 The qualitative data were analyzed using content analysis. This method is commonly used in the analysis of text data.

 Meta-analytical statistical methods were used to estimate the variables of the Ki-67 index rate. The meta-analysis was carried out using STATA software version 16. The results were presented using forest plot diagrams. The heterogeneity of the study results was assessed using Q and I^2^. The Higgins et al^28^ thresholds were used to evaluate the I^2^ (25% for low heterogeneity, 50% for medium, and 75% for high heterogeneity). The random model was selected due to the high heterogeneity of the research. In addition, subgroup analyses based on data sources (patient document review/laboratory sample) were carried out. Finally, the funnel plot diagram and Begg’s regression test were used to quantify publication bias at a significance level of 0.01.

## Results

**Figure 1:**
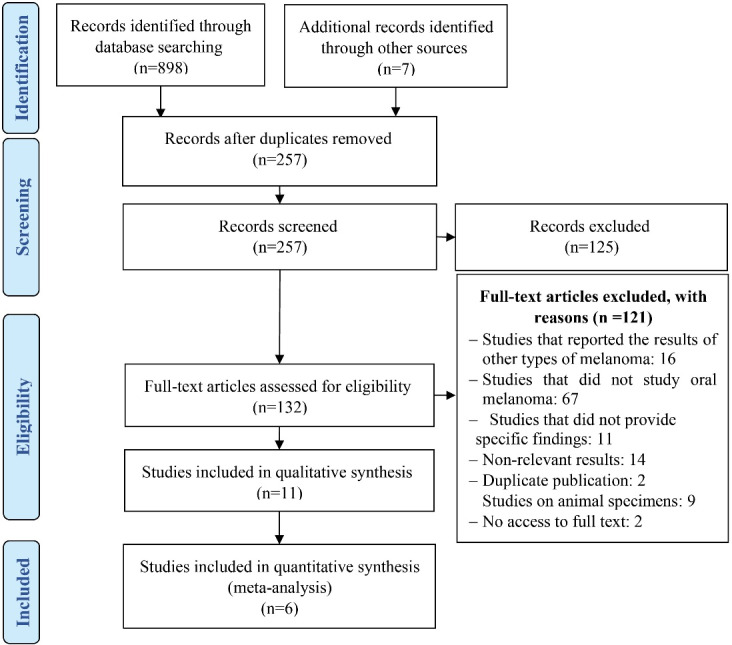
Searching an1d screening process

 Totally 905 articles were retrieved in the literature search process. After removing duplicates, articles were screened based on title and abstract. About 132 articles were reviewed for eligibility, and 11 articles were included (Table 1), of which 6 articles were meta-analyzed. The study screening and selection process are reported in the PRISMA flow diagram (Figure 1).

**Table 1:** Detail information about included studies that investigated the effect of the Ki-67 index in the diagnosis of oral melanoma

**Authors, Years**	**Study Design**	**Sample Size**	**Age (y)**	**Ki-67 expression (%)**	**Overall survival (months)**	**Results**
Soares et al,^24^ 2020	Patient record review	7	51.5	Amelanotic: 64 (34‒92)	-	-
		22	58	Melanotic 30.8 (11‒59)		
Rodrigues et al,^4^ 2021	Patient record review	7	58	70 (n = 5) 90 (n = 2)	-	-
Rivera et al,^29^ 2007	Patient record review	19	-	-	-	Ki-67 was widely expressed by melanoma cells
Perri et al,^30^ 2017	Patient record review	20	54	-	-	the proliferation marker Ki-67 was higher in patients with poor outcomes, and all the patients surviving at 24 months had a low expression of this marker
Ma et al,^31^ 2017	Patient record review	123	54	n = 27 → (<5)	1.59	- There were more Ki-67-positive cells in nodular lesions than in macular lesions (*P*<0.001). - Ki-67 expression was associated with tumor type in OMM. - The expression of Ki-67 in recurrent OMM was significantly higher than in primary OMM (*P* = 0.03). - Cases with high Ki-67 expression had significantly poorer overall survival. - Ki-67 expression was an independent prognostic factor for poor overall survival - OMM patients with higher Ki-67 showed poor survival time in comparison to those with lower Ki-67
				n = 19→ (5-10)	2.59	
				n = 26→ (10-20)	3.50	
				n = 14 → (20-30)	4.38	
				n = 24 → (30-40)	5.38	
				n = 13→ (>50)	6.37	
Korabiowska et al,^32^ 2005	Patient record review	6 without metastases	67	18 (4‒29)	-	The Ki-67 index expression differed significantly between melanomas with and without metastases (*P*<0.05)
		11 with metastases		47 (8‒79)		
		12 with metastases lymph nodes and organs		68.6 (12‒95)		
de-Andrade et al,^33^ 2012	Laboratory sample	22	58	23.7 (15.51‒63)	-	-
de-Andrade et al,^3^ 2013	Laboratory sample	11	23-86	42.5 (15.5‒65)		-
Buery et al,^34^ 2010	Patient record review	19	58	>80		- Ki-67 was only detected at the epithelial basal layer in oral MM (oral melanotic macule) and was completely negative in nevus cells. - No gender predilection was observed.
Alaeddini and Etemad-Moghadam,^1^ 2014	Patient record review	19	59	22.09 ± 15.88		-
Zhu et al^35^ 2015	Patient record review	9	-	≥ 30 (n = 5) <30 (n = 4)		Patients with anorectal melanoma had less survival time than patients with an oral cavity (*P* = 0.025)

 Meta-analysis was run based on data from 307 individuals. Most of the included studies (11 studies) were published until April 2022. Detailed information about included studies is presented in Table 1.

###  Ki-67 index rate

**Figure 2:**
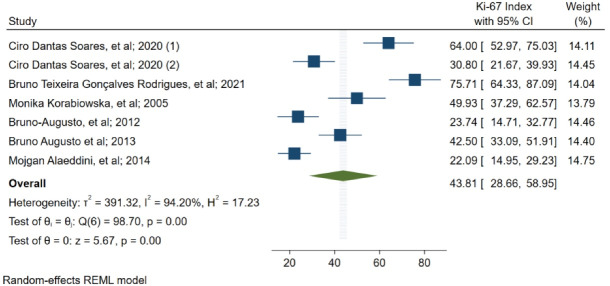
Ki-67 index rate in patients with oral melanoma

 Ki-67 index in patients with oral melanoma was reported by 6 studies (Figure 2). This index was estimated at 43.81 (28.66–58.95 with 95% CI, I^2^ = 94.2). The results revealed a high level of heterogeneity among study results. Moreover, publication bias test results showed that the probability of publication bias in the results was 0.016 (Publication Bias-Begg’s test, *P* = 0.016).

###  Ki-67 index rate based on subgroups

**Figure 3:**
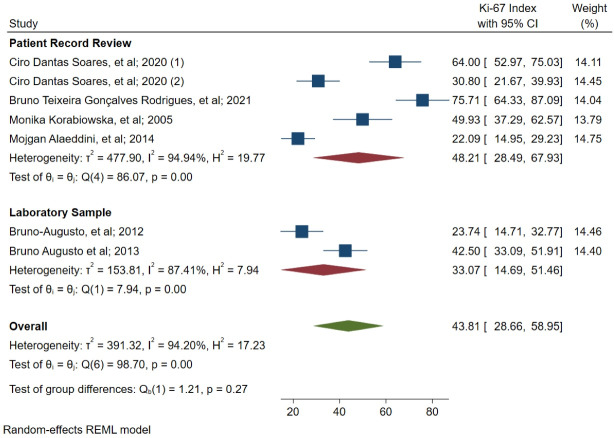
Ki-67 index rate in patients with oral melanoma based on subgroups

 A meta-analysis of the difference between the patient record review and laboratory sample studies was performed based on data source subgroups (Figure 3). Four studies estimated ki-67 based on patients’ records and two studies based on laboratory samples. The index inpatient record review was estimated at 48.21 (28.49–67.93 with 95 CI, I^2^ = 94.94) and laboratory sample studies at 33.07 (14.69‒51.46 with 95 CI, I^2^ = 87.41). The results revealed no significant difference between the two groups (*P* = 0.27).

###  Quality appraisal results

 The average overall quality of reporting in the articles was 31.6 (rang = 0-36). The reporting quality of included articles was estimated as a good category, generally.

###  Effect of Ki-67 index expression on the prognosis of oral melanoma

 Studies have shown that the marker Ki-67 is widely expressed in melanoma cells. Some studies have shown that in oral melanotic macule lesions, this marker is expressed only in the epithelial basal layer and is not expressed in nevus cells. There was no difference in expression between men and women. Also, the studies extracted in this study showed a significant effect between increasing the expression of this marker and decreasing the prognosis of oral melanoma.

 Ki-67-positive cells were reported in nodular lesions more than macular lesions. Ki-67 expression is associated with the type of malignant oral melanoma tumor. For example, the expression of Ki-67 in recurrent OMM was significantly higher than that in primary OMM. As a result, OMM patients with higher Ki-67 had a shorter survival time than those with lower Ki-67.

## Discussion

 This systematic review and meta-analysis was performed to estimate the Ki-67 index in patients with oral melanoma based on 11 studies on 307 patients. Ki-67 index in patients with oral melanoma was estimated at 43.81 (28.66–58.95 with 95% CI, I^2^ = 94.2). Subgroup meta-analysis results showed that the Ki-67 index rates in patient record review and laboratory sample studies were 48.21 (28.49–67.93 with 95% CI, I^2^ = 94.94) and 33.07 (14.69–51.46 with 95% CI, I^2^ = 87.41), respectively. The extracted studies also showed a significant relationship between the expression of the Ki-67 marker and the prognosis of oral melanoma. Patients with metastases to lymph nodes and other body organs showed higher expression of this marker. The survival rate of these patients was significantly lower, and they had a poor prognosis.

 Some studies showed that Ki-67 expression was an independent prognostic factor for poor overall survival.^31^ Some other studies have shown that the expression of Ki-67 also increases with metastasis to different body organs. Patients without metastasis had an average Ki-67 index rate of 18. In contrast, those with metastases to lymph nodes and other organs had an average Ki-67 index rate of 68.6.^32^ Ki-67-positive cells were more common in nodular lesions than macular lesions in nodular melanoma.^31^ Ki-67 expression was significantly higher in recurrent oral mucosal melanoma than in the primary.^31^

 The meta-analysis showed higher Ki-67 factor levels in patient record studies than in laboratory sample research. The overall meta-analysis result for the Ki-67 index rate was 43.81 (28.66‒58.95 with 95% CI, I^2^ = 94.2). According to a study on cutaneous melanoma, tumors that expressed more Ki-67 (more than 10%) were thicker than tumors that expressed lower levels of this marker.^17^ Another study comparing the expression of this marker in patients with and without metastasis showed that in patients with oral melanoma with metastasis to various body organs, the average Ki-67 marker was 68%, which is about 60% higher than in patients without metastasis.

 Based on the inclusion and exclusion criteria, we finally selected 11 articles, 6 of which were chosen for meta-analysis. However, 5 studies could not be included in the meta-analysis. Some of them, for example, did not have any quantitative data that could be used to calculate the Ki-67 marker expression index,^29,30^ and in others, some quantitative data were reported based on cut-off point values.^31,35^ Also, some studies have shown the Ki-67 marker expression without mentioning their means.^33,34^ In some studies, the sample size was not classified based on metastasis. However, one study divided its sample size into metastatic and non-metastatic sections^32^; therefore, we calculated the weighted mean to be able to include that study in the meta-analysis.

 The expression level of the Ki-67 marker in oral melanoma is an effective factor in determining the prognosis of this disease. According to this systematic review, individuals with oral melanoma with higher expression of this marker have a lower survival rate. There was also a significant relationship between this marker and the spread of metastasis to other body organs.^32^

 According to several long-term follow-up studies, the Ki-67 marker could be used to classify some cancer patients, including those with breast cancer.^13,36^ In addition, several studies have shown an association between Ki-67 expression and the thickness of cutaneous melanoma lesions, as well as a relationship between Ki-67 expression and the tumor’s long-term prognosis.^19^ However, the effect of this marker on the long-term prognosis of mucosal melanoma requires further studies.

 Unfortunately, since quantitative data for prognosis was not collected, a meta-analysis of this factor was not possible. As a result, the impact of this factor on the prognosis of lesions cannot be quantified. To collect accurate quantitative data on the effect of the Ki-67 marker on the prognosis of oral melanoma, further research should be conducted to examine the Ki-67 index and the survival of these patients. Further research is necessary to determine the Ki-67 index expression threshold for lesion metastasis. In addition, staging the Ki-67 index expression level to evaluate the prognosis of the lesion can be a useful in determining the prognosis of oral melanoma lesions and developing an effective treatment plan for this illness.

## Conclusion

 The expression of the Ki-67 factor is higher in individuals with oral melanoma than in healthy people. As this marker’s expression rises, so will the spread of metastasis to other organs of the body, and as metastasis increases, so will the disease severity. Therefore, this marker can be effective in the diagnosis and prognosis of oral melanoma.

## Author Contributions

 MM and PM were mainly responsible for the design and supervision of the study. MM and SP were involved in the article screening. RR performed the meta-analysis. MM, PM and RR prepared the manuscript, and all the authors revised and approved it.

## Funding

 This study has no funding source.

## Ethics Approval

 This study was part of an approved study by the Research Ethics Committee of the Tabriz University of Medical Sciences (No: IR.TBZMED.REC.1401.074).

## Competing Interests

 There are no conflicts of interest relevant to this article.
